# Open-circuit diagnosis method based on dual-mode voltage residual model for T-type three-level inverter

**DOI:** 10.1038/s41598-025-98747-w

**Published:** 2025-04-23

**Authors:** Liming Song, Minxuan Liao, Rongkun Wang, Xinhua Guo

**Affiliations:** College of Information Science and Engineering, Huaqiao University, Xiamen, 361001 China

**Keywords:** Three-level fault-tolerant algorithm, Line voltage residual, Fault diagnosis, T-type three-level inverter, Electrical and electronic engineering, Hydroelectricity

## Abstract

This study proposes a rapid online diagnostic method based on a dual-mode line voltage residual model for diagnosing IGBT open-circuit faults in a T-type three-level inverter. The method is based on the voltage residual method and is integrated with a three-level fault-tolerant algorithm. Firstly, by analyzing the line voltage residuals after an open-circuit fault in the T-type inverter under normal operating conditions, 12 types of single-switch open-circuit faults are divided into 6 groups. Next, the breakdown of the spatial voltage vectors following the fault are analyzed, and the remaining vectors are reconfigured to enable the inverter’s operation under three-level fault-tolerant control. In the fault-tolerant control, the residual voltage returns to its normal value, aiding in precise fault localization. Simulation and experimental results validate the method’s robustness against load variations. This method significantly increases diagnostic speed and reduces harmonic interference in the diagnosis procedure. These findings are of paramount importance for fault diagnosis and the reliable operation of the T-type three-level inverter.

## Introduction

Multi-level inverters are widely used in medium and low voltage applications with moderate switching frequencies^1,2^, such as renewable energy systems and electric vehicles. The T-type three-level (T^2^3L) inverter is among the most commonly employed multi-level inverters due to its simple structure, ease of control^3^, high transmission efficiency^4^, and exceptional fault tolerance performance^5^.

Fault diagnosis and fault-tolerant control (FTC) are critical for ensuring the reliable and stable operation of the T^2^3L inverter^6^. The FTC refers to the ability of the inverter to output a normal waveform after a fault, by reassigning the remaining healthy switches. In the event of a single-switch open-circuit (OC) fault in the power switches, appropriate allocation of the remaining switches can enable fault-tolerant operation. This can minimize the detrimental impact on the entire system. Conventional fault diagnostic methods and the fault-tolerant control are studied separately. This is because not all inverters can achieve fault-tolerant control, so the research on fault diagnosis often focuses solely on fault location without addressing subsequent fault-tolerant control. However, the T-type three-level inverter is capable of FTC. After a single-switch failure occurs, it can continue to operate through the intervention of a fault-tolerant algorithm. This increases its operational reliability. So, the fault diagnosis is first conducted to locate the fault position, and then corresponding fault-tolerant control is performed. However this approach inevitably prolongs the duration of the fault state and has a certain impact on the load. Generally, the protective circuit integrated into the drive board can detect short-circuit (SC) faults of power devices^7,8^. Through the SC protection circuit, a drive signal is immobilized to convert it into an OC fault or to be promptly isolated^9^, thus averting further damage. In the case of OC faults, current diagnostic methods primarily analyze voltage and current signals to extract fault features for fault localization^10,11^.

Voltage and current residuals^12–14^ are commonly utilized in OC fault diagnosis for various inverters because they can rapidly indicate the degree of waveform distortion. In Ref.^15^, the residual error of the current in a permanent magnet synchronous motor-driven inverter was analyzed, focusing on diagnosing 21 OC faults in a 6-phase two-level inverter by examining the magnitude and characteristics of the current residual error. In Ref.^16^, the Parker transform was applied to the phase voltage residual to identify OC faults in a three-phase bridge inverter. This method ensures fast diagnosis and high reliability. In Ref.^17^, the Parker transformation was applied to the output current of the permanent magnet synchronous motor drive inverter to obtain the current trajectory on the plane. The trajectory residual is then obtained and fault diagnosis is performed based on the angle and size of the residual vector. Additionally, a hybrid logic dynamic model of the motor drive system was developed to estimate the motor current, thereby reducing the sensitivity of the current to load variations.

However, these diagnostic methods have been primarily developed for two-level inverters. In the two-level inverter, each bridge arm contains only one switching device, allowing faults in different bridge arms to be distinguished by observing the direction of change in the voltage or current residuals. But in the case of T-type three-level inverters, which incorporate a large number of switches in each bridge arm, the analysis of circuit becomes significantly intricate. Accurately pinpointing the fault location using solely one type of voltage or current poses a significant challenge. Generally, a combination of other signal characteristics is necessary to achieve precise fault localization. For instance, in Ref.^18^, the authors utilized the phase voltage residuals of an APF inverter to classify fault characteristics. They combined these characteristics with current residuals to enhance the accuracy of fault localization. This method showcases rapid diagnostic speed, albeit it may exhibit sensitivity to load variations. To address this concern, Ref.^19^ employed the residual of DC-side capacitor voltages instead of current residuals for fault diagnosis, thereby improving its robustness. However, the variation of capacitor voltage remains slow and limited under the midpoint balancing suppression strategy. In Ref.^20^, the polarity of current residuals in different sectors was analyzed following a fault occurrence in the T-type inverter, enabling initial fault differentiation. Subsequently, the inverter modulation coefficient is controlled at 0.25. Then the variation of the voltages in DC-bus was used to achieve OC fault localization with high reliability. Nevertheless, the diagnostic time exceeds one fundamental cycle. To mitigate the influence of the midpoint balancing strategy on capacitor voltage suppression and the sensitivity of current to load, Ref.^21^ proposed a two-step mechanism for fault diagnosis in the T-type inverter. In the first step, fault detection is performed using phase voltage residual vectors, and the fault types are preliminarily grouped based on the phase and magnitude of the residual vectors. In the second step, the inverter is transitioned from a three-level mode to a two-level mode, enabling precise localization using phase voltage residuals once again. This diagnostic method demonstrates strong robustness and high reliability. However, the three-level inverter fails to fully exploit redundant switching vectors in the two-level mode, resulting in reduced efficiency. To achieve efficient fault-tolerant operation, it becomes necessary to switch back to the three-level mode after completing the diagnosis. So it has a complex process and requires further improvement in diagnostic time.

In this paper, we propose a two-step diagnostic method based on a dual-mode line voltage residual model. The method analyzes line voltage residuals and integrates a three-level FTC algorithm. It enables fast fault localization (group-level localization) in the normal mode by analyzing the line voltage residual. On the other hand, considering that the goal of fault diagnosis for the T-type inverter is FTC, the FTC algorithm is preemptively introduced upon detecting the fault to achieve a second fault localization (precise fault localization) under the mode of FTC. This effectively reduces the process of fault diagnosis-tolerant control. Moreover, precise fault localization under three-level fault-tolerant control significantly reduces harmonics caused by waveform distortion during the fault, minimizing damage to the load. The effectiveness of the proposed method is demonstrated through both simulation and experimental results.

## Fault analysis in the normal mode of T-type three level inverter

**Fig. 1:**
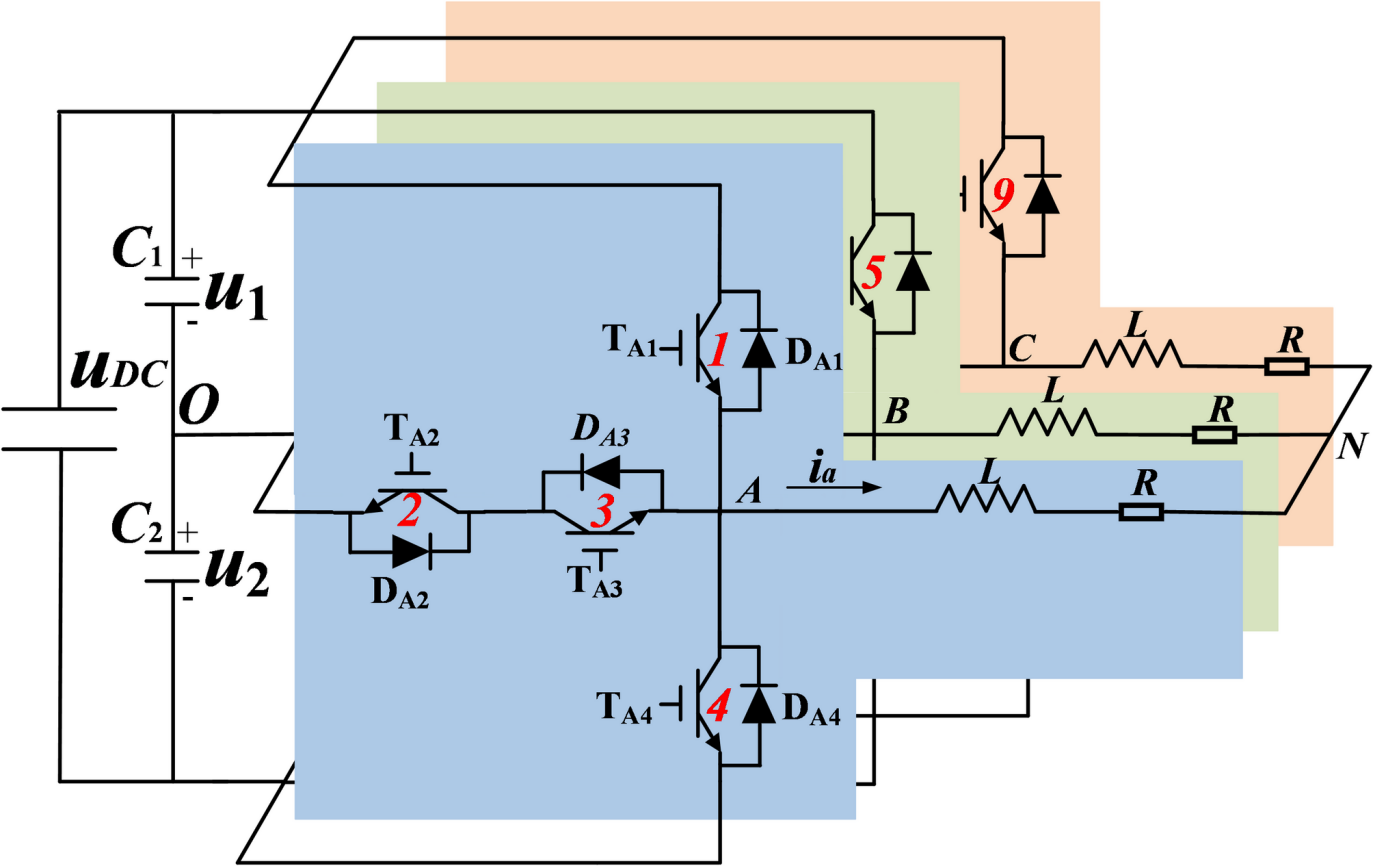
Basic topology of T-type three-level inverter.

Figure 1 illustrates the basic the fundamental topology of a T^2^3L inverter, which includes two capacitors on DC-side with voltages denoted as *u*_*1*_ and *u*_*2*_, and their values are $\:{u}_{DC}/2$. Each phase consists of four IGBTs and their antiparallel diodes, which are vertical arm (T_X1_,T_X4_) and horizontal arm (T_X2_,T_X3_) respectively. For ease of subsequent analysis, the power switches T_A1_, T_A2_…T_C3_ and T_C4_ are numbered from 1 to 12 successively as in Fig. 1. As an illustrative example under typical motor driving conditions, the load exhibits three-phase symmetry.

There are three bridge output states for each phase during normal operation, namely P, O and N. The corresponding switch states and voltage amplitudes are shown in Table 1. In Table 1, 0 and 1 represent the off and on states of the switch, respectively.

**Table 1:** Basic switch status and output voltage.

T_A1_	T_A2_	T_A3_	T_A4_	Output state	Voltage
1	0	1	0	P	$\:{u}_{DC}/2$
0	1	1	0	O	0
0	1	0	1	N	$\:-{u}_{DC}/2$

For ease of subsequent analysis, we clearly define the switch functions corresponding to the three output states at the bridge terminals during normal operation as *S*_*X*_:

1 $${S_X}=\left\{ {\begin{array}{*{20}{c}} {1,({T_{X1}},{T_{X3}}=on,{T_{X2}},{T_{X4}}=off)} \\ {0,({T_{X2}},{T_{X3}}=on,{T_{X1}},{T_{X4}}=off)} \\ { - 1,({T_{X2}},{T_{X4}}=on,{T_{X1}},{T_{X3}}=off)} \end{array}} \right.(X=A,B,C)$$

In (1), X represents the phase sequence. *S*_*X*_ ={1,0,-1}, it corresponds to the output voltage of $\:-{u}_{DC}/2$, 0, and $\:{u}_{DC}/2$, respectively. The voltage of the inverter bridge terminals relative to the neutral point O is:

2 $${u_{XO}}=\frac{{{u_{DC}}}}{2} \cdot {S_X}$$

In (2), *u*_*DC*_ is the voltage of the DC bus. The phase voltages can be expressed as:

3 $${u_{XN}}={u_{XO}}+{u_{ON}}$$

Meanwhile, due to the three-phase symmetry, we have:

4 $${u_{AN}}+{u_{BN}}+{u_{CN}}=0$$

By combining (1)-(4), we can obtain:

5 $$\left[ {\begin{array}{*{20}{c}} {{u_{AB}}} \\ {{u_{BC}}} \\ {{u_{CA}}} \end{array}} \right]=\frac{{{u_{DC}}}}{2}\left[ {\begin{array}{*{20}{c}} {{S_A} - {S_B}} \\ {{S_B} - {S_C}} \\ {{S_C} - {S_A}} \end{array}} \right]$$

For ease of subsequent analysis, the values of voltage residual are defined as $\:\varDelta\:{u}_{X}$:

6 $$\Delta {u_X}=u - {u^*}$$

In (6), *u* represents a actual output voltage, and u* represents a desired output voltage, which can be predicted by the current trigger signals of switches.

**Fig. 2:**
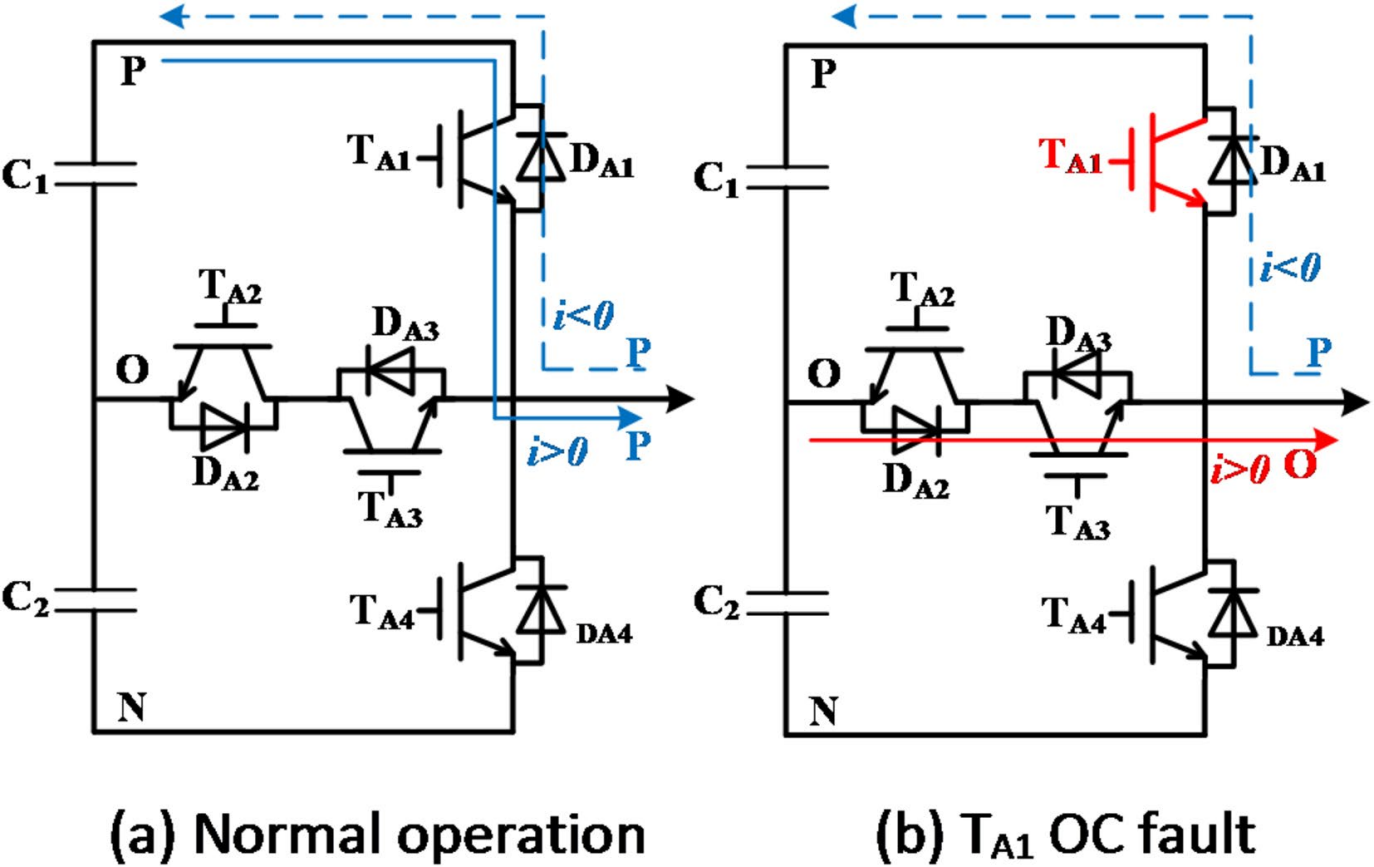
The change of output state after T_A1_ fails.

The load exhibits symmetry, with phase-A serving as an example. Figure 2 illustrates the circuit state transition before and after the OC fault occurs in T_A1_ of the vertical bridge arm. As depicted in Fig. 2, it is evident that following the occurrence of an OC fault in T_A1_, if the current direction is negative, the output voltage at the bridge terminal will change from the P state to the O state. The residuals of the three-phase line voltages are calculated based on (5) and (6), and the result is shown in (7).

7 $$\left[ {\begin{array}{*{20}{c}} {\Delta {u_{AB}}} \\ {\Delta {u_{BC}}} \\ {\Delta {u_{CA}}} \end{array}} \right]=\frac{{{u_{DC}}}}{2}\left[ {\begin{array}{*{20}{c}} {{S_A} - S_{A}^{*}} \\ {{S_B} - S_{B}^{*}} \\ {S_{A}^{*} - {S_A}} \end{array}} \right]=\left[ {\begin{array}{*{20}{c}} { - \frac{{{u_{dc}}}}{2}} \\ 0 \\ {\frac{{{u_{dc}}}}{2}} \end{array}} \right]$$

**Fig. 3:**
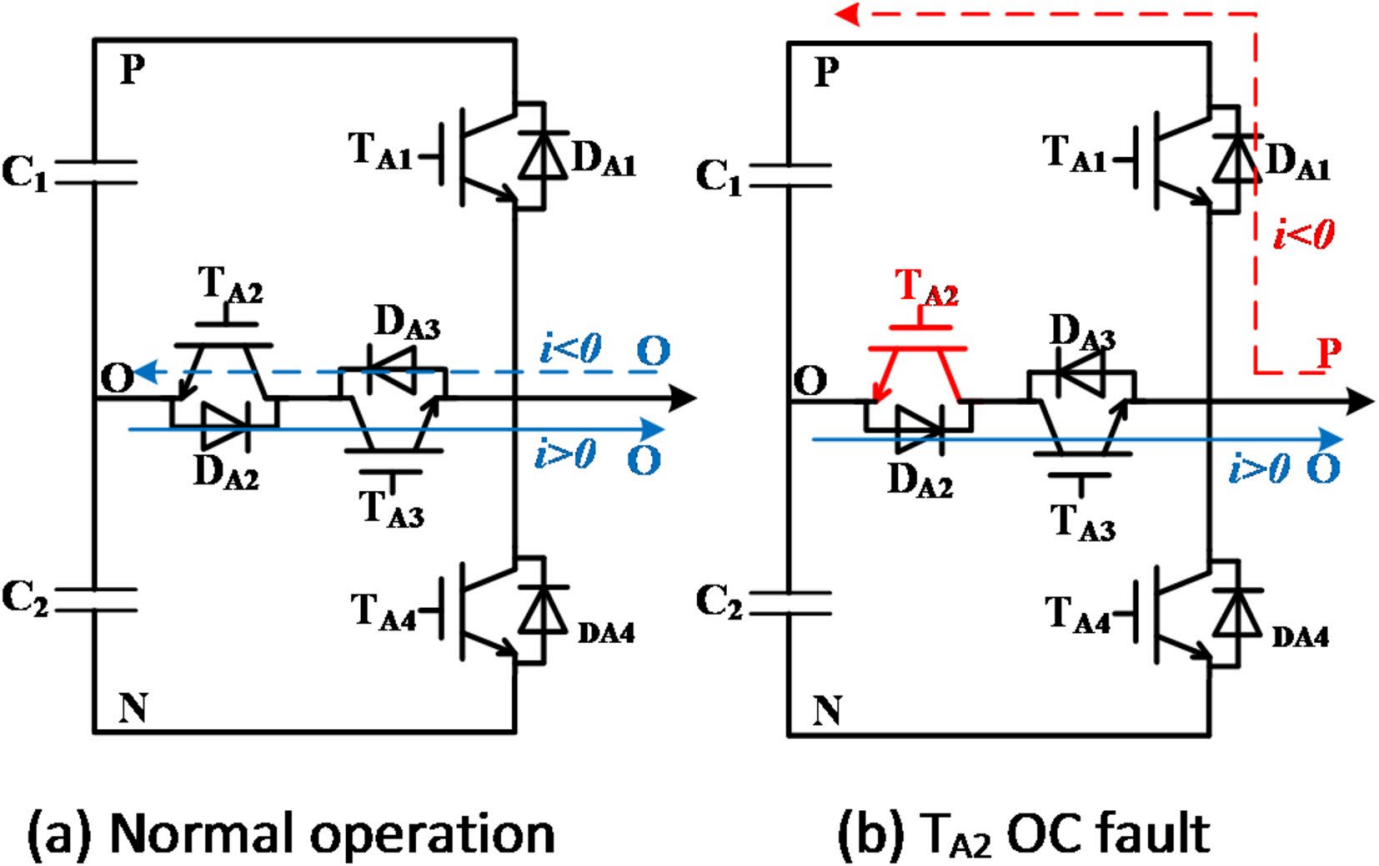
The change of output state after T_A2_ fails.

As the OC fault of T_A2_, the output state will change as well during half of the current cycle, as shown in Fig. 3.

Similarly, the residuals of three-phase line voltages are calculated as (8):

8 $$\left[ {\begin{array}{*{20}{c}} {\Delta {u_{AB}}} \\ {\Delta {u_{BC}}} \\ {\Delta {u_{CA}}} \end{array}} \right]=\frac{{{u_{DC}}}}{2}\left[ {\begin{array}{*{20}{c}} {{S_A} - S_{A}^{*}} \\ {{S_B} - S_{B}^{*}} \\ {S_{A}^{*} - {S_A}} \end{array}} \right]=\left[ {\begin{array}{*{20}{c}} {\frac{{{u_{dc}}}}{2}} \\ 0 \\ { - \frac{{{u_{dc}}}}{2}} \end{array}} \right]$$

The residuals of three-phase line voltages after the OC fault of T_A3_ are shown in (9); The residuals of three-phase line voltages after the OC fault of T_A4_ are shown in (10).

9 $$\left[ {\begin{array}{*{20}{c}} {\Delta {u_{AB}}} \\ {\Delta {u_{BC}}} \\ {\Delta {u_{CA}}} \end{array}} \right]=\left[ {\begin{array}{*{20}{c}} { - \frac{{{u_{dc}}}}{2}} \\ 0 \\ {\frac{{{u_{dc}}}}{2}} \end{array}} \right]$$

10 $$\left[ {\begin{array}{*{20}{c}} {\Delta {u_{AB}}} \\ {\Delta {u_{BC}}} \\ {\Delta {u_{CA}}} \end{array}} \right]=\left[ {\begin{array}{*{20}{c}} {\frac{{{u_{dc}}}}{2}} \\ 0 \\ { - \frac{{{u_{dc}}}}{2}} \end{array}} \right]$$

In ideal conditions, the actual value of output voltage matches the desired value, resulting in zero residuals for line voltage. By monitoring any changes in the residuals of line voltage, a failure can be detected. The formula used for determining such failures is as follows:

11 $$\gamma =\left\{ {\begin{array}{*{20}{c}} {1,\left| {\Delta {u_X}} \right| \geqslant TH} \\ {0,\left| {\Delta {u_X}} \right|<TH} \end{array}} \right.\left( {X=AB,BC,CA} \right)$$

Equation (11) uses 0 and 1 to represent normal and fault states, respectively, with TH indicating the trigger threshold. As per (5), residual values of voltage are solely dependent on the DC-bus voltage and switch state, and remain unaffected by the load. When the switch function is constant, the line voltages are only related to the bus voltage. Typically, the bus voltage change will not exceed 5%. To prevent false diagnosis caused by sampling noise and detection margin, a threshold of TH = 10%$\:\cdot\:{u}_{DC}$ can be used. The summary of changing residuals of line voltages for 12 different faults are shown in Table 2.

**Table 2:** Detection criteria in the normal mode.

Faults	$\:{\gamma\:}_{1}$	$\:{\gamma\:}_{2}$	$\:{\gamma\:}_{3}$
Normal	0	0	0
T_A1_ or T_A3_	-1	0	1
T_A2_ or T_A4_	1	0	-1
T_B1_ or T_B3_	1	-1	0
T_B2_ or T_B4_	-1	1	0
T_C1_ or T_C3_	0	1	1
T_C4_ or T_C4_	0	-1	-1

For ease of subsequent analysis, we introduce a fault diagnosis variable *F*_*1*_, to represent the condition of the inverter:

12 $${F_1}=({\gamma _1},{\gamma _2},{\gamma _3})$$

As shown in Table 2, a normal operation is indicated by *F*_*1*_ = (0,0,0), while any other value indicates a fault condition. By examining voltage residuals, it is possible to identify a faulty phase and the parity of the number related to the power switches; however, localization of a single switch cannot be accomplished with accuracy. The next section will analyze the voltage residuals during three-level FTC mode.

## Fault analysis in the fault-tolerant mode of T-Type inverter

### Three-level fault-tolerant control

**Fig. 4:**
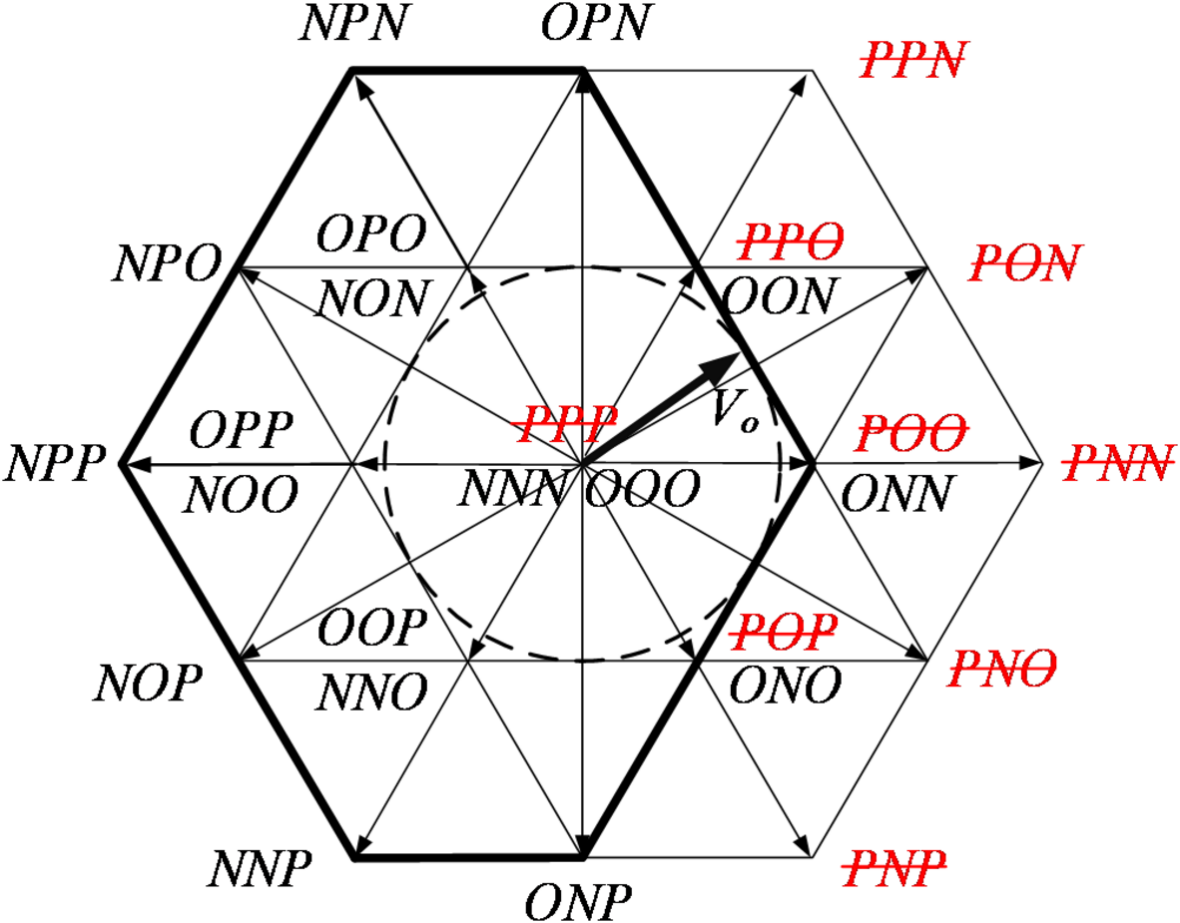
The distribution of space voltage vectors after T_A1_ fails.

**Fig. 5:**
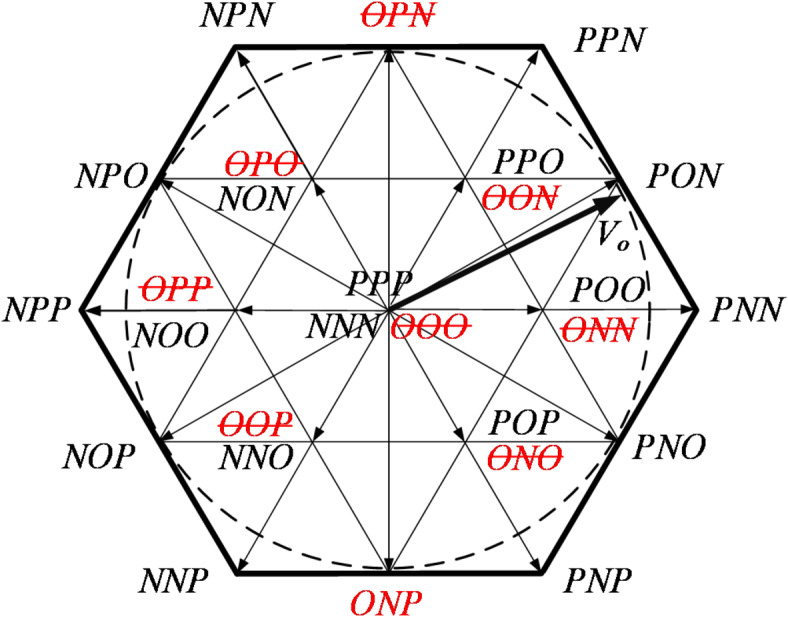
The distribution of space voltage vectors after T_A2_ fails.

The T-type three-level inverter, with its greater number of switches, enables FTC in the event of a single-switch fault. In the FTC mode, the inverter’s operation is managed by the remaining healthy switches, ensuring the symmetry of the output voltage waveform and achieving high energy transmission efficiency, thereby significantly contributing to the reliable operation of T-type three-level inverters. The T^2^3L inverters can be controlled using the seven-segment SVPWM algorithm. In three-level mode, the algorithm has a total of 27 basic spatial voltage vectors. As detailed in Sect. 2, different switching failures lead to distinct changes in the output state, which correspond to varying failures of spatial voltage vectors. The following takes the A-phase T_A1_ and T_A2_ switches as examples to analyze the distribution of spatial voltage vectors after the OC fault, as shown in Figs. 4 and 5, respectively.

After the failure of T_A1_, the state P will change to O. There are 9 basic vector failures, corresponding to the red vectors in Fig. 4. The long vectors PPN, PNN, and PNP have no alternative vectors, while the small vectors PPO, POO, POP, and the zero vector PPP can be replaced by redundant vectors. For example, PPO, POO, POP and PPP can be replaced by OON, ONN, ONO, and OOO, respectively. By confining the output range to the black dashed circle in Fig. 4, the synthesized reference vector can still achieve a relatively standard circular trajectory.

After the failure of T_A2_, two medium-length vectors (OPN and ONP) fail. Among them, OPN can be replaced by PPN or NPN, and ONP can be replaced by NNP or PNP. All six short vectors and the zero vector have at least one effective redundant switch state. After the fault occurs, there is no need to reduce the output, as three-phase symmetric operation can still be ensured.

**Fig. 6:**
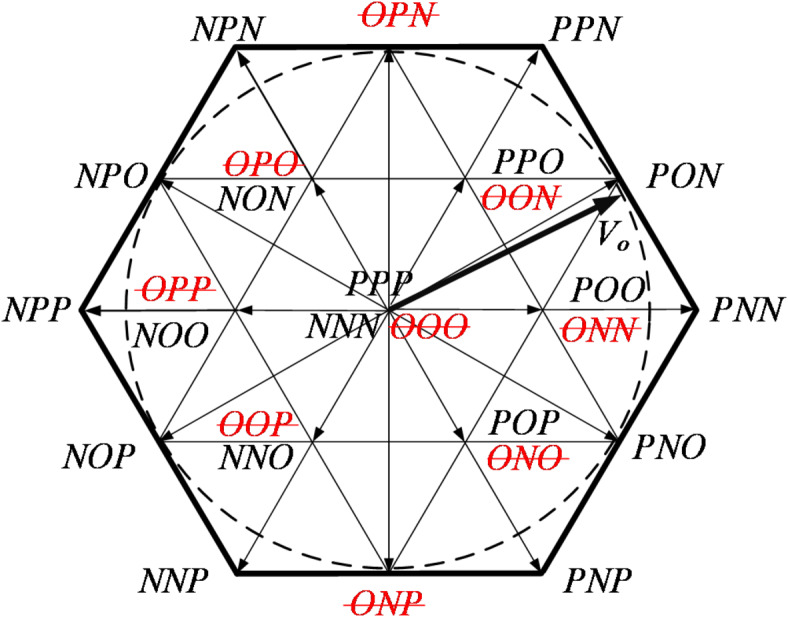
The distribution of space voltage vectors after T_A3_ fails.

**Fig. 7:**
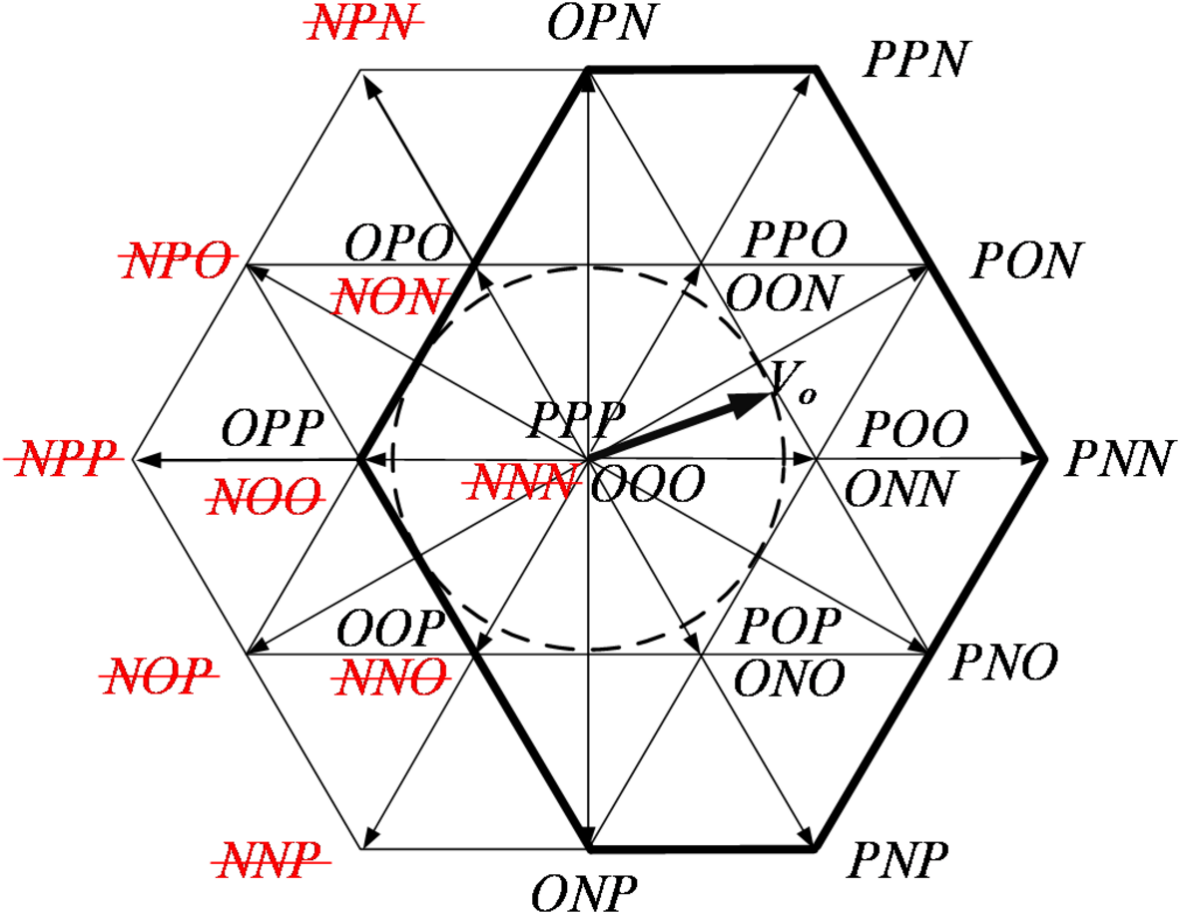
The distribution of space voltage vectors after T_A4_ fails.

After the OC of T_A3_ and T_A4_, the distribution of spatial voltage vectors is shown in Figs. 6 and 7, respectively. It is evident that when the vertical bridge arm (T_A1_, T_A4_) fails, the effective range of voltage vectors reduces, and the modulation factor of the SVPWM algorithm can still operate within a range less than 0.5. On the other hand, when the horizontal bridge arm (T_A2_, T_A3_) fails, the effective range of voltage vectors remains unchanged, and the system can still operate fault-tolerantly with a modulation factor greater than 0.5.

### Residual voltage characteristic extraction under the FTC mode

For ease of subsequent analysis, the FTC algorithm corresponding to each switch after failure is defined as FTC_X (X = 1,2,3…12), following the order of switch numbering. In the three-level FTC mode, the fault diagnosis variable *F*_*2*_ is defined as (13) to extract the fault characteristics.

13 $${F_2}=({\gamma _1},{\gamma _2},{\gamma _3})$$

The value of each element in *F*_*2*_ continues to be calculated according to (11). In a correct FTC mode, the employed space voltage vectors are all healthy redundant vectors. Therefore, the theoretical difference between the expected output voltage and the actual voltage should be zero. It is evident that in the FTC_3 mode, regardless of whether T_A2_ or T_A3_ fails, *F*_*2*_=(0,0,0) always holds, whereas when T_A1_ or T_A4_ fails, *F*_*2*_≠(0,0,0) always holds. The B and C phases exhibit similar behavior, which can aid in further fault localization. When the horizontal bridge arms (T_A2_, T_A3_) experience the same fault, their corresponding fault-tolerant algorithms are identical. Therefore, we define FTC_A = FTC_3; FTC_B = FTC_7; FTC_C = FTC_11, and the criteria for three-level FTC are presented in Table 3.

**Table 3:** Detection criteria in the FTC mode.

Faults	FTC_A mode	FTC_B mode	FTC_C mode
None	*F*_*2*_=(0,0,0)	*F*_*2*_=(0,0,0)	*F*_*2*_=(0,0,0)
T_A1_ or T_A4_	*F*_*2*_≠(0,0,0)	–	–
T_A2_ or T_A3_	*F*_*2*_=(0,0,0)	–	–
T_B1_ or T_B4_	–	*F*_*2*_≠(0,0,0)	–
T_B2_ or T_B3_	–	*F*_*2*_=(0,0,0)	–
T_C1_ or T_C4_	–	–	*F*_*2*_≠(0,0,0)
T_C2_ or T_C3_	–	–	*F*_*2*_=(0,0,0)

## The diagnostic method of dual-mode voltage residual model

**Fig. 8:**
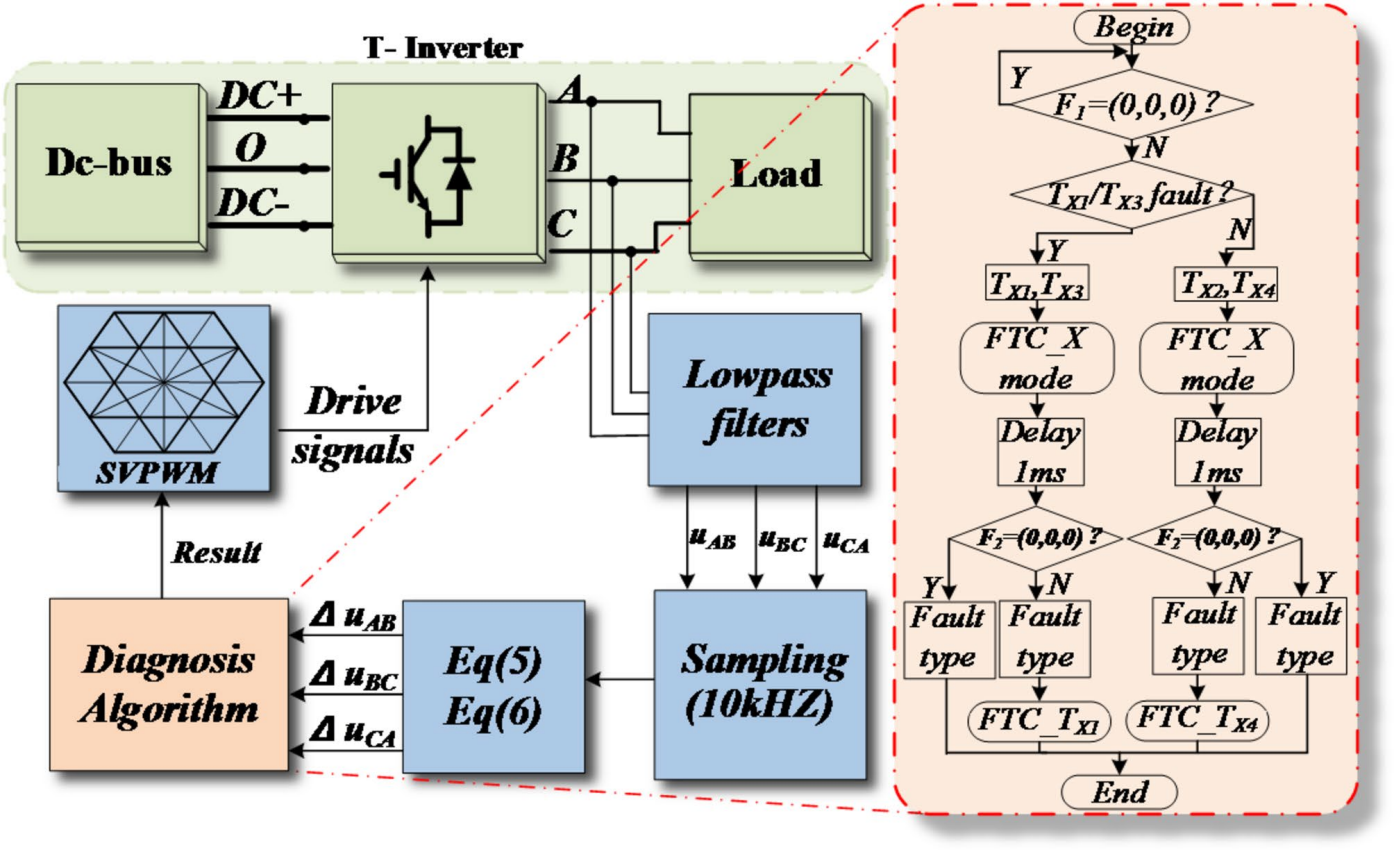
The waveforms of three-level tolerant control.

Through the analysis of Sections III and IV, it is evident that the residual values of line voltages in normal and FTC modes vary depending on which switch has failed. Based on this, a fault diagnostic strategy based on the dual-mode line voltage residual model is proposed. Figure 8 presents a comprehensive diagnostic flowchart.

## Simulation and experiment

### Simulation validation of the proposed FTC algorithm

To validate the effectiveness of the three-level fault-tolerant control algorithm, a simulation model is constructed. In the simulation, the trigger pulses of T_A1_ and T_A2_ fail at 0.06s, respectively, to simulate the OC fault. To facilitate the observation of fault-tolerant effects, the fault-tolerant algorithm is applied after 30ms.

**Fig. 9:**
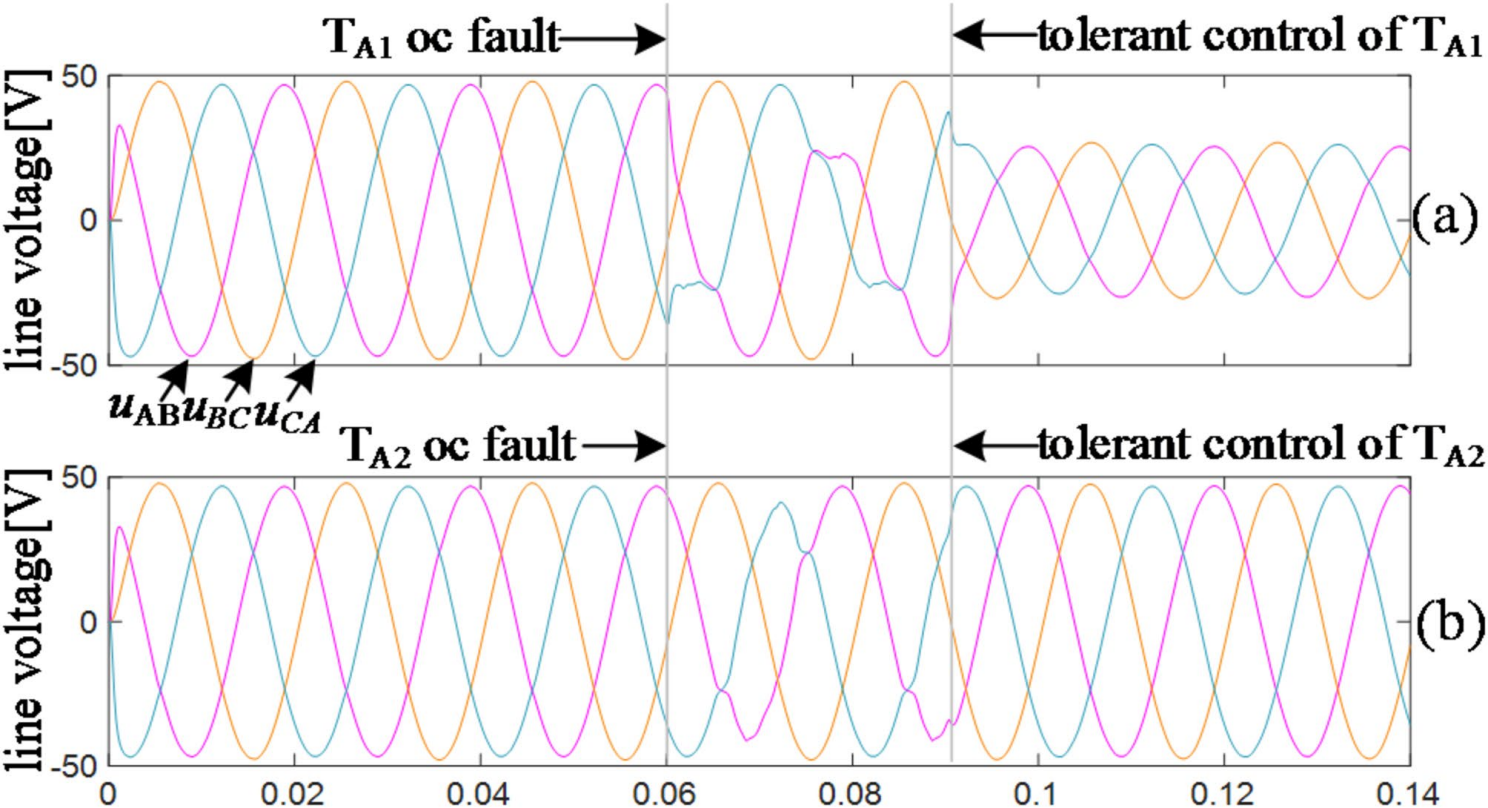
The waveforms of three-level tolerant control.

The results are presented in Fig. 9 (a-b). As depicted in Fig. 9 (a), when the vertical bridge arm T_A1_ fails, the inverter can still maintain symmetric operation by derating the output. As illustrated in Fig. 9 (b), when the horizontal bridge arm T_A2_ fails, there is no need to reduce the output, and the inverter can still achieve symmetric operation, thereby aligning with the aforementioned theory.

### Simulation validation of the proposed fault diagnostic method

To verify the effectiveness of the proposed method, a simulation model of T^2^3L inverter is constructed and the parameters in the simulation are shown in Table 4.

**Table 4:** Parameters in simulation.

Parameter	Value
DC voltage (*u*_*dc*_)	60 V
Three phase inductance (*L*)	3 mH
Switching frequency (*f*_*s*_)	10 kHz
DC side capacitances (*C*)	*C*_1_ = *C*_2_ = 4700µF
Three-phase resistance *(R)*	8 /16 Ω
Output frequency (fR)	50 Hz

**Fig. 10:**
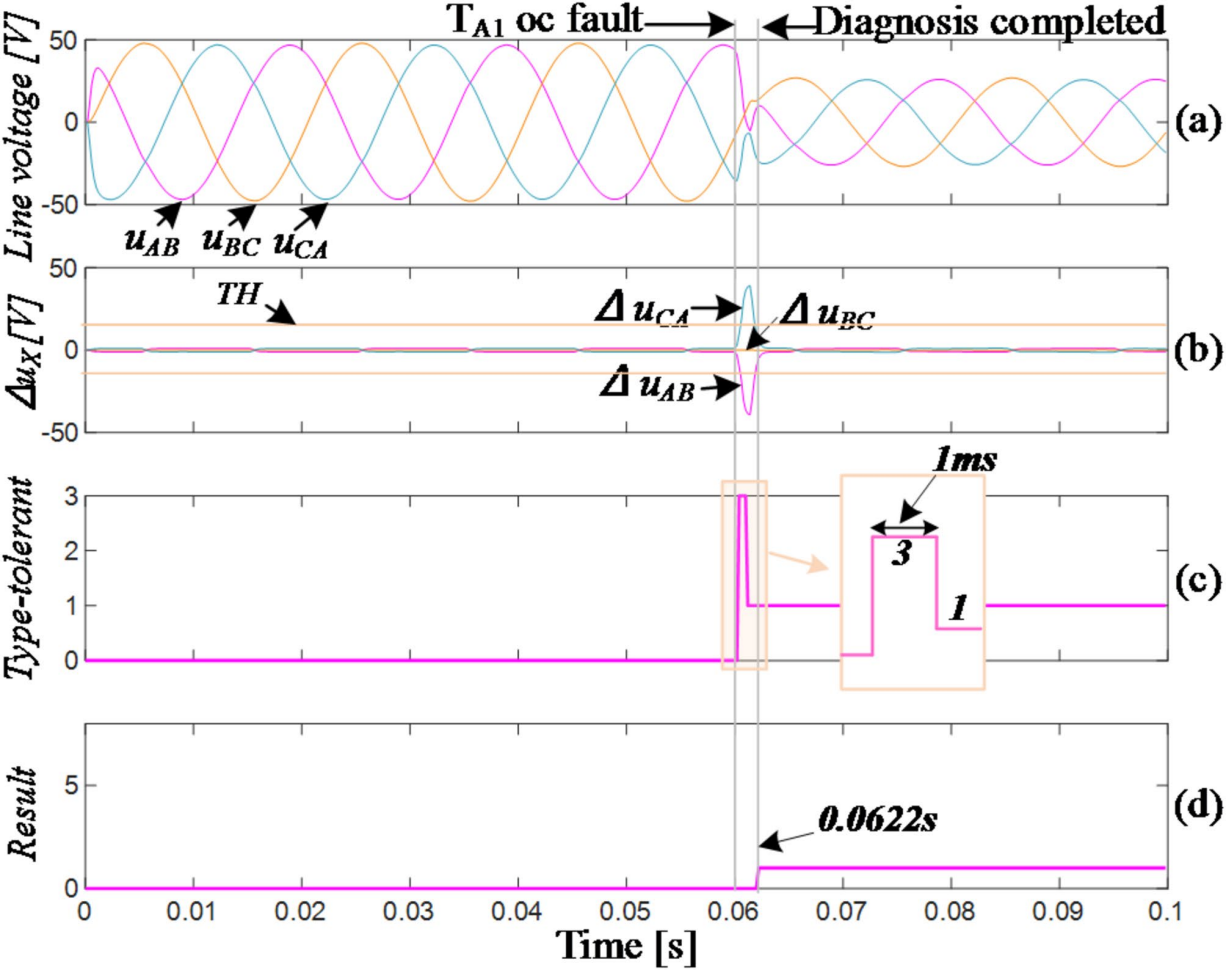
Simulation of T_A1_ fails.

The waveforms captured during simulation of the open circuit in the vertical bridge arm of T_A1_ are displayed in Fig. 10. T_A1_ failed at 0.06s. The residuals of *u*_*CA*_ and *u*_*AB*_ exceeded the positive and negative thresholds, respectively, while the residual of *u*_*CA*_ remained within the normal range. Therefore, the fault vector *F*_*1*_=(-1,0,1) was obtained, indicating an odd-number switching failure in the A-phase (T_A1_/T_A3_). The inverter immediately transitions to FTC_3 mode. After a 1ms delay, the three-phase voltage values do not return to the normal range, indicating that *F*_*2*_≠(0,0,0) and corresponding to an open-circuit (OC) fault of T_A1_. The T_A1_ fault-tolerant control algorithm (FTC_1) is utilized to ensure that the voltage residuals return to normal values with no misdiagnosis. The entire diagnosis process took 2.2ms, approximately 1/10 of a fundamental cycle. In the event of a T_A3_ fault occurring at the same moment, the fault location can be determined within 1ms after entering FTC_3 mode for the first time, reducing diagnostic time by 1ms, or approximately 1.2ms.

**Fig. 11:**
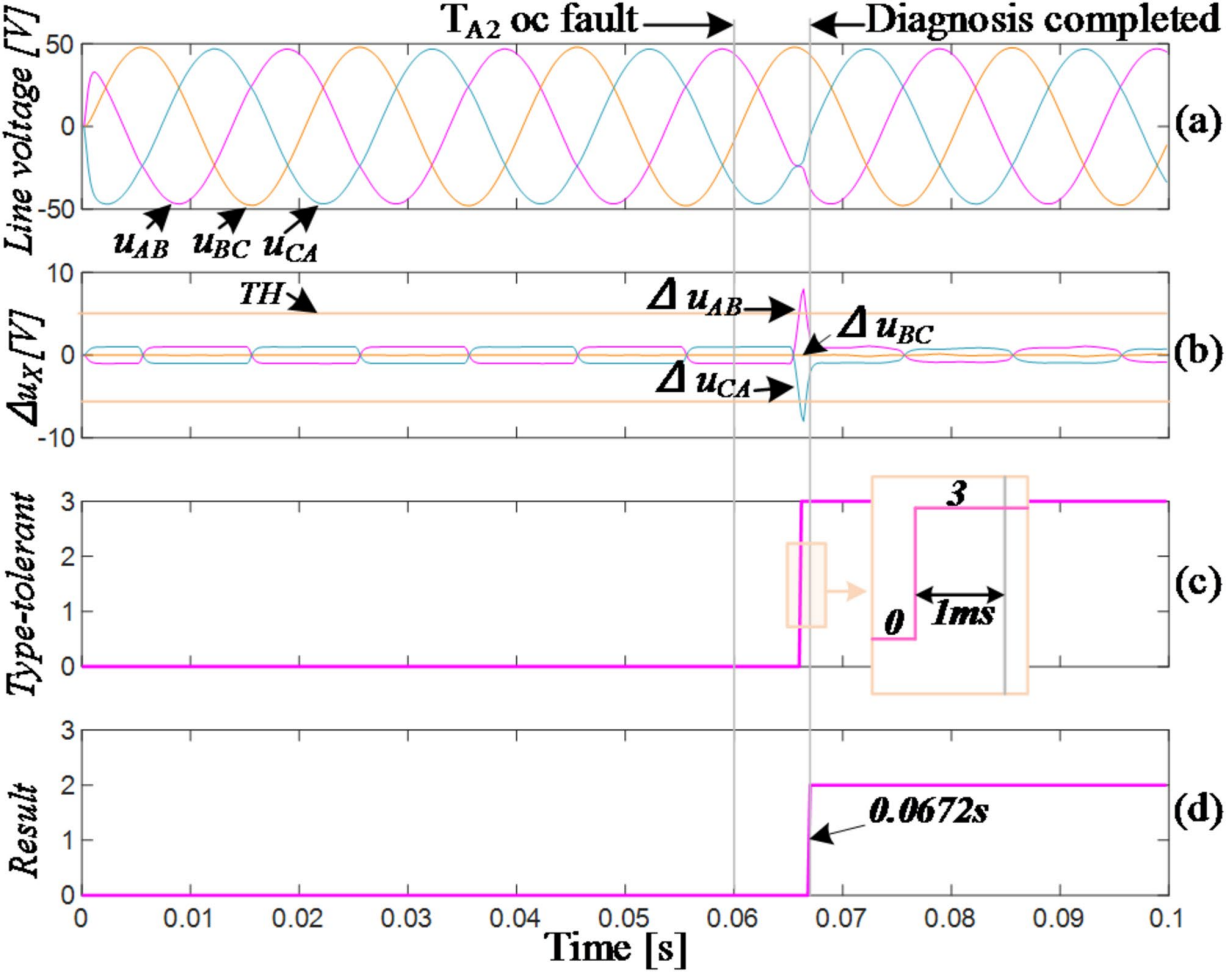
Simulation of T_A2_ fails.

Figure 11 shows a failure of T_A2_ at 0.06s. Analysis of the phase relationship between the line voltage and line current indicates that at this time, the current direction in the A-phase is towards the bus capacitor from the load side. Based on Fig. 3, the circuit state does not change immediately. Once the current direction changes, the residual values of line voltages *u*_*AB*_ and *u*_*CA*_ exceed the thresholds in positive and negative directions, respectively, while the residual value of *u*_*BC*_ remains within a safe range. This corresponds to *F*_*1*_=(1,0,-1), indicating a fault in the A-phase (T_A2_/T_A4_). The circuit immediately enters into FTC_3 mode. After a 1ms delay, the residual values of the three-phase line voltages returned to safe ranges, which is indicated by *F*_*2*_=(0,0,0). The diagnosis was confirmed to be completed with a total time of 7.2ms, approximately 1/3 of the fundamental period.

### Experimental validation of the proposed fault diagnostic method’s effectiveness

**Fig. 12:**
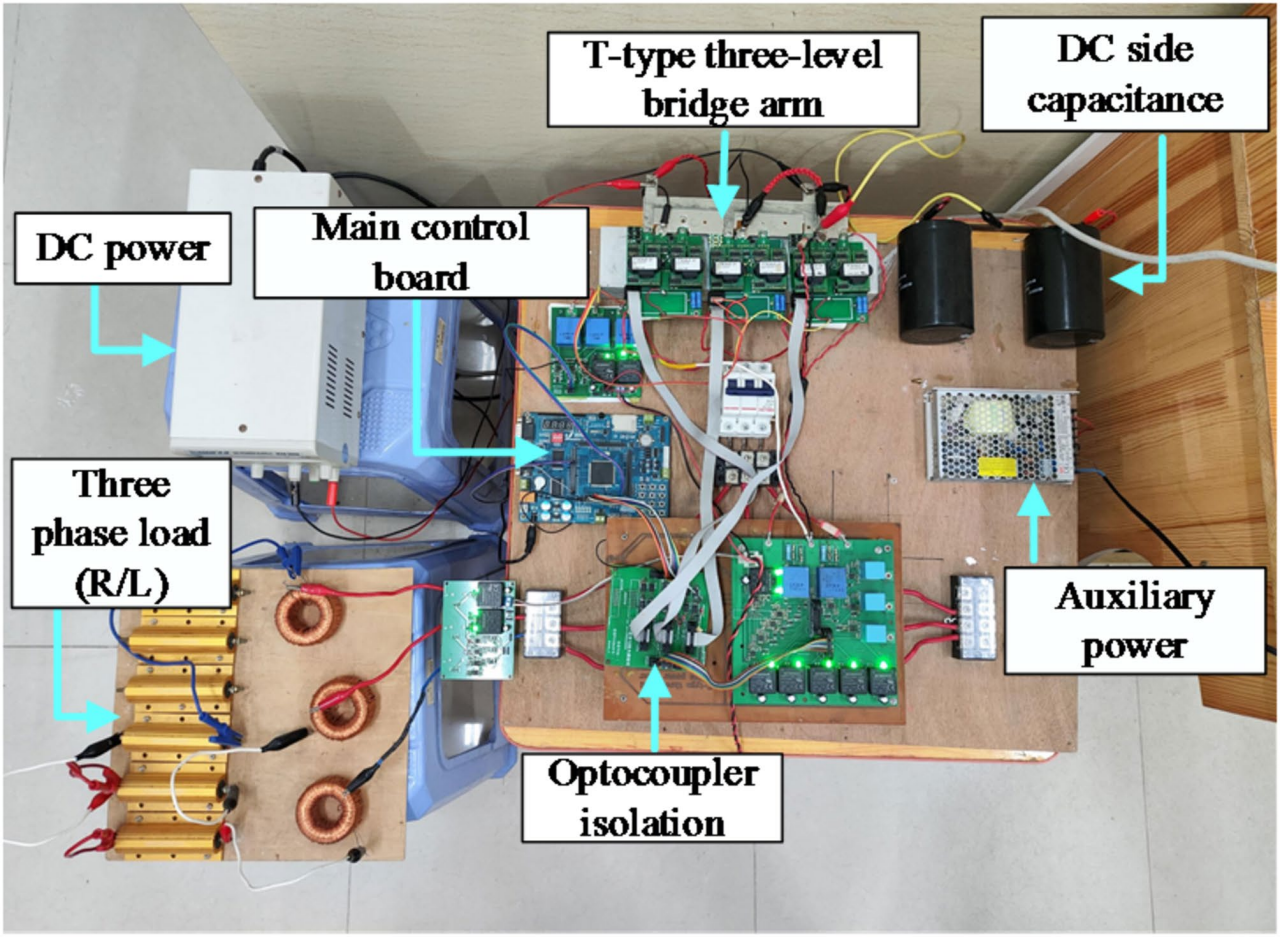
T-type three-level inverter hardware platform.

To further confirm the effectiveness of the proposed method, a hardware platform for the T^2^3L inverter was constructed, as depicted in Fig. 12. For the experiment, the main control device utilizes the DSP28335 from TI, using a seven segment SVPWM algorithm. The pulse waves are isolated by the optocoupler and transmitted to the driving circuit. The driving circuit connect to the T-type topology. The driving circuit amplifies the pulse signal and drives the IGBT to conduct and turn off. The signal during the experiment is collected through a sampling circuit, and the experimental data is sent to the PC every 500us through serial communication. The core device models of each part are: Optocoupler isolation chip is ACPL-P480; The voltage sensor of the sampling circuit is LV-25P; The three-phase T-type bridge arm are three IGBT modules, F3L100R12W2H3_B11; The driving circuit are the 2SC0108T2G0-17 module. Other parameters not mentioned are consistent with Table 4. And MATLAB is used to redraw the waveforms.

**Fig. 13:**
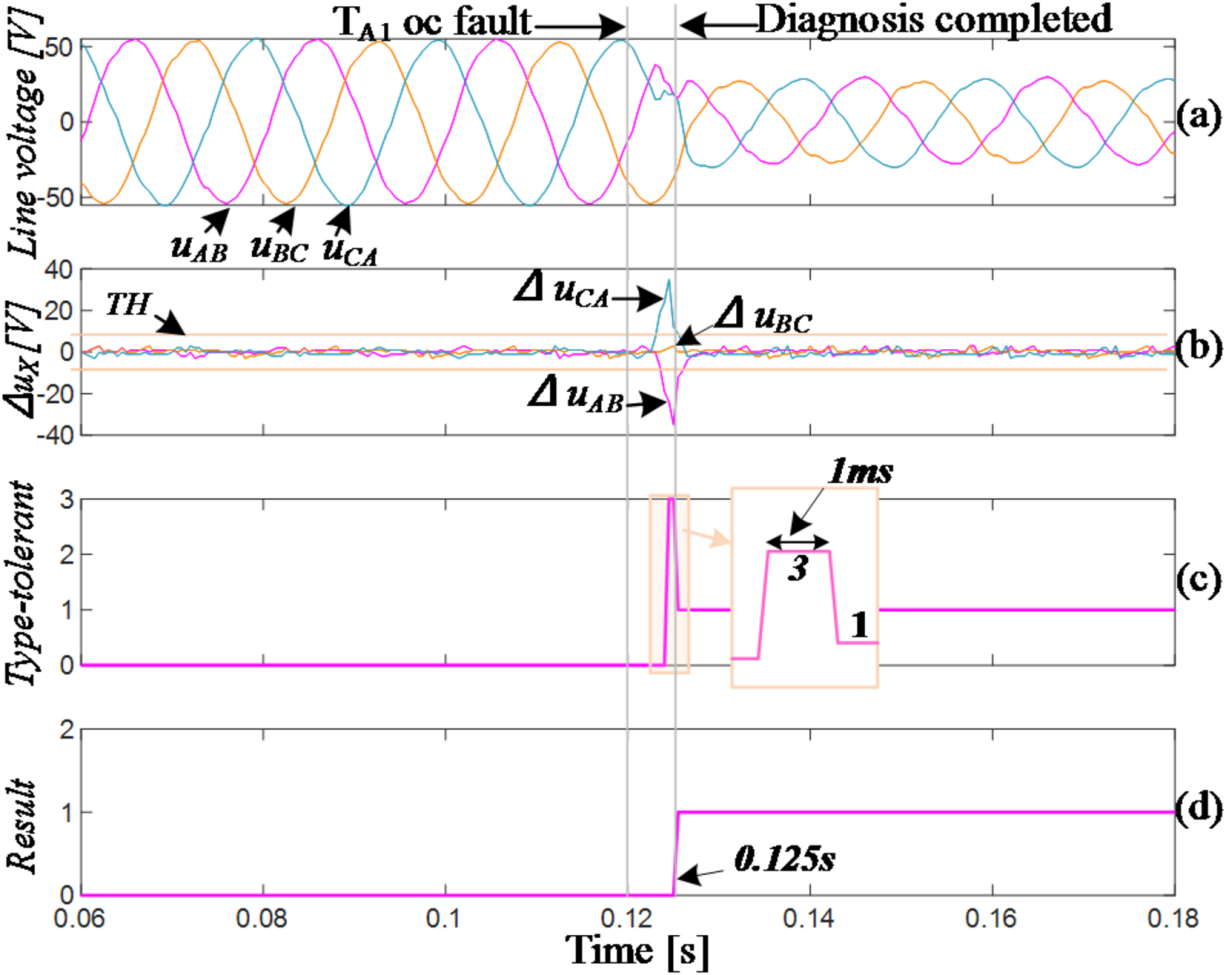
Experiment of T_A1_ fails.

**Fig. 14:**
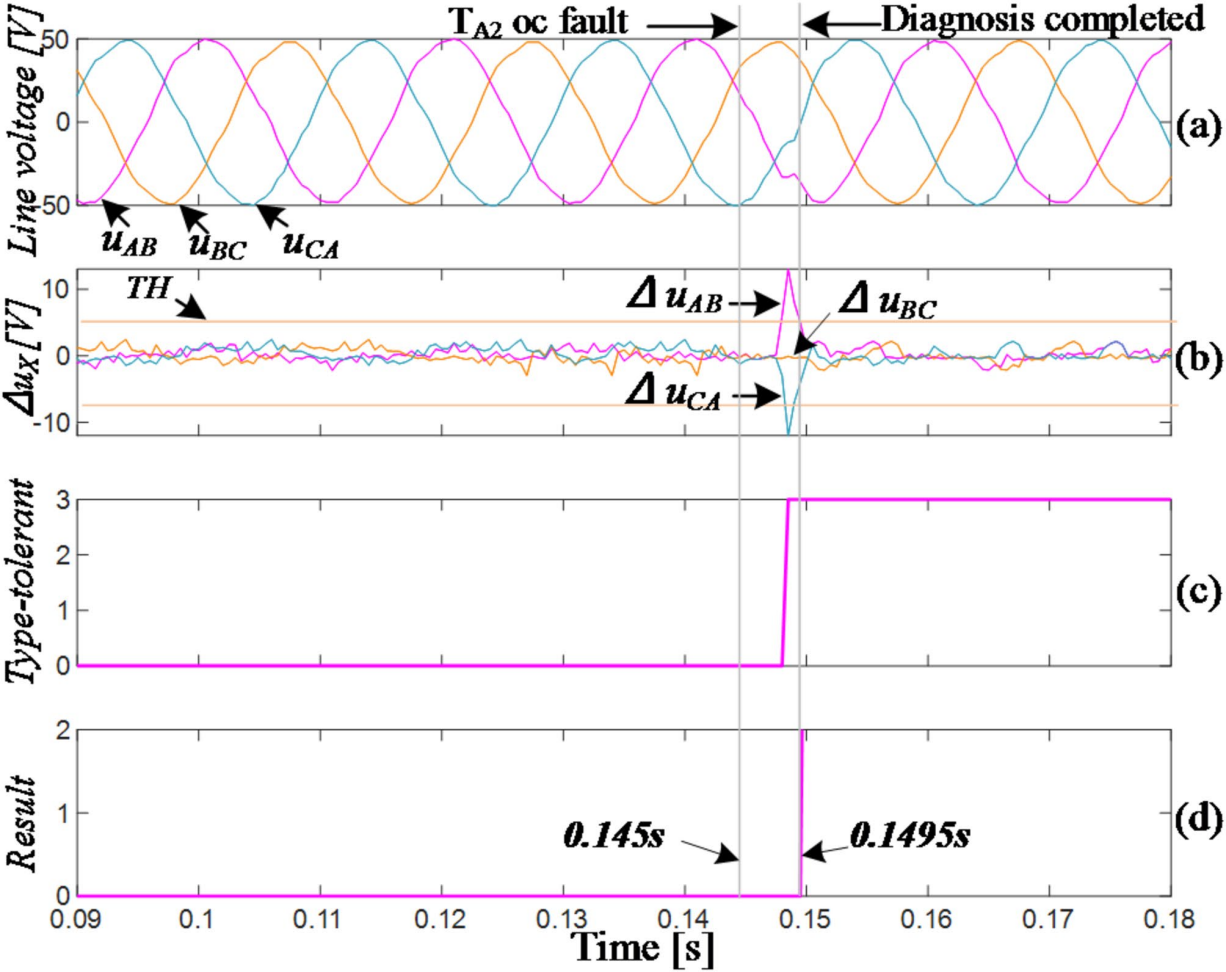
Experiment of T_A2_ fails.

Figures 13 and 14 show the waveforms of T_A1_ and T_A2_ OC faults in the experiment, respectively.

Compared to the simulated waveforms, the residual values of the three-phase line voltages exhibit larger fluctuations in normal conditions, but they still remain below the diagnostic threshold. In Fig. 13, T_A1_ fails at approximately 0.12s. The residual values of line voltages *u*_*CA*_ and *u*_*AB*_ exceeding the threshold in positive and negative directions respectively, corresponding to *F*_*1*_=(-1,0,1). Subsequently, the inverter changes to the FTC mode. Through two changes of fault-tolerant type, the OC fault of T_A1_ is located. It takes about 5ms. In the FTC_1 mode, the waveforms of the three-phase line voltages are slightly distorted compared to the simulated waveforms but maintain a sinusoidal state overall.

Due to the improved output capability during fault-tolerant operation after a horizontal bridge arm fault, the overall waveforms in Fig. 14 exhibit little distortion before and after the T_A2_ fault. T_A2_ fails at approximately 0.145s, followed by the residual values of line voltages *u*_*AB*_ and *u*_*CA*_ exceeding the threshold in positive and negative directions respectively, corresponding to *F*_*1*_=(1,0,-1). The inverter then immediately changes into the FTC_3 mode. In the mode, the output line voltages return to the normal condition immediately, and after 1ms, the fault of T_A2_ is identified, with a total time of approximately 4.5ms. Additionally, the output voltage amplitude remains largely unchanged, and the output capability is still comparable to the normal state, indicating a better fault-tolerant effect.

### Robustness verification of the proposed fault diagnostic method

**Fig. 15:**
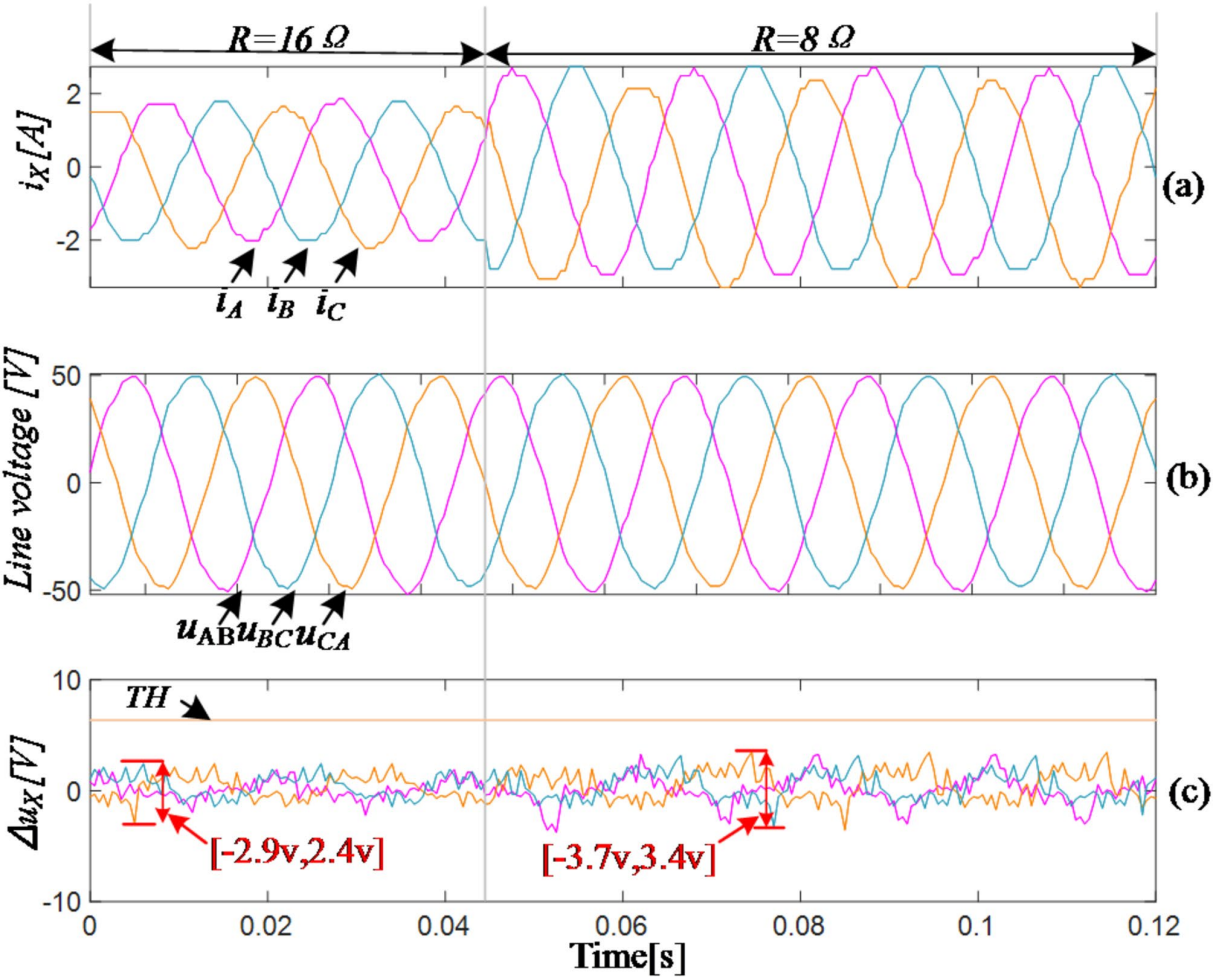
Experiment of load variation.

The waveforms change of the primary diagnostic variables are displayed in Fig. 15 when the load changes from 16Ω to 8Ω. The subfigures a and b show the waveforms of three-phase current and three-phase line voltage, respectively. And subfigure c shows the residual values of the three-phase line voltage. As can be seen in Fig. 15, at the instant of load change, the three-phase currents undergo significant sudden variations, while the line voltages of three-phase remain relatively unchanged. The residual values of line voltages increase slightly, and the voltages change range before the load change is [-2.9 V,2.4 V], while after the load is halved, the change range is [-3.7 V,-3.4 V], which is slightly wider, but still within acceptable limits, indicating that the proposed method has a high robustness.

### Performance analysis and comparison with other diagnostic methods

Table 5 presents the simulation data pertaining to the open-circuit diagnostic time and the accuracy rate. The first three columns present data related to diagnostic time, while the last column displays the accuracy of the diagnostic algorithm. These data are derived from 100 simulation experiments, with the fault occurrences uniformly distributed throughout a single current cycle.

**Table 5:** Statistics results of diagnostic time.

Faulty switch	$\:{t}_{min}$/ms	$\:{t}_{max}$/ms	$\:{t}_{aver}$/ms	Accuracy rate
T_A1_	2.1ms	12.2ms	6.9ms	99%
T_A2_	1.2ms	11.3ms	6.4ms	99%

These data are derived from 50 simulation experiments, with the fault occurrences uniformly distributed throughout a single current cycle. As shown in the table, the fastest diagnostic time for a horizontal switch fault is 2.1 ms, while the slowest is 12.2 ms. Based on the circuit theory analysis presented in Sect. 2, it is established that regardless of which switch fails, there will be instances when the output state remains unchanged for half a current cycle. Consequently, the maximum diagnostic time will exceed half of the fundamental cycle. Notably, for horizontal bridge arm faults, the average diagnostic time is significantly reduced. This is because, after a horizontal bridge arm fault, the fault-tolerant algorithm requires only a single switch operation to achieve fault localization. For ease of subsequent analysis, we reference the signal’s fundamental period, Tac. The fastest diagnostic time for an open-circuit fault in the horizontal bridge arm (T_A2_) is 1.2 ms, equivalent to 0.06Tac. In contrast, the slower diagnostic time for an open-circuit fault in the vertical bridge arm (T_A1_) is 12.2 ms, or 0.61Tac. On the other hand, as indicated by the data in the last column, the fault location accuracy reached 99% in 100 simulation trials. It indicates that the proposed fault diagnosis method has a high accuracy rate.

**Fig. 16:**
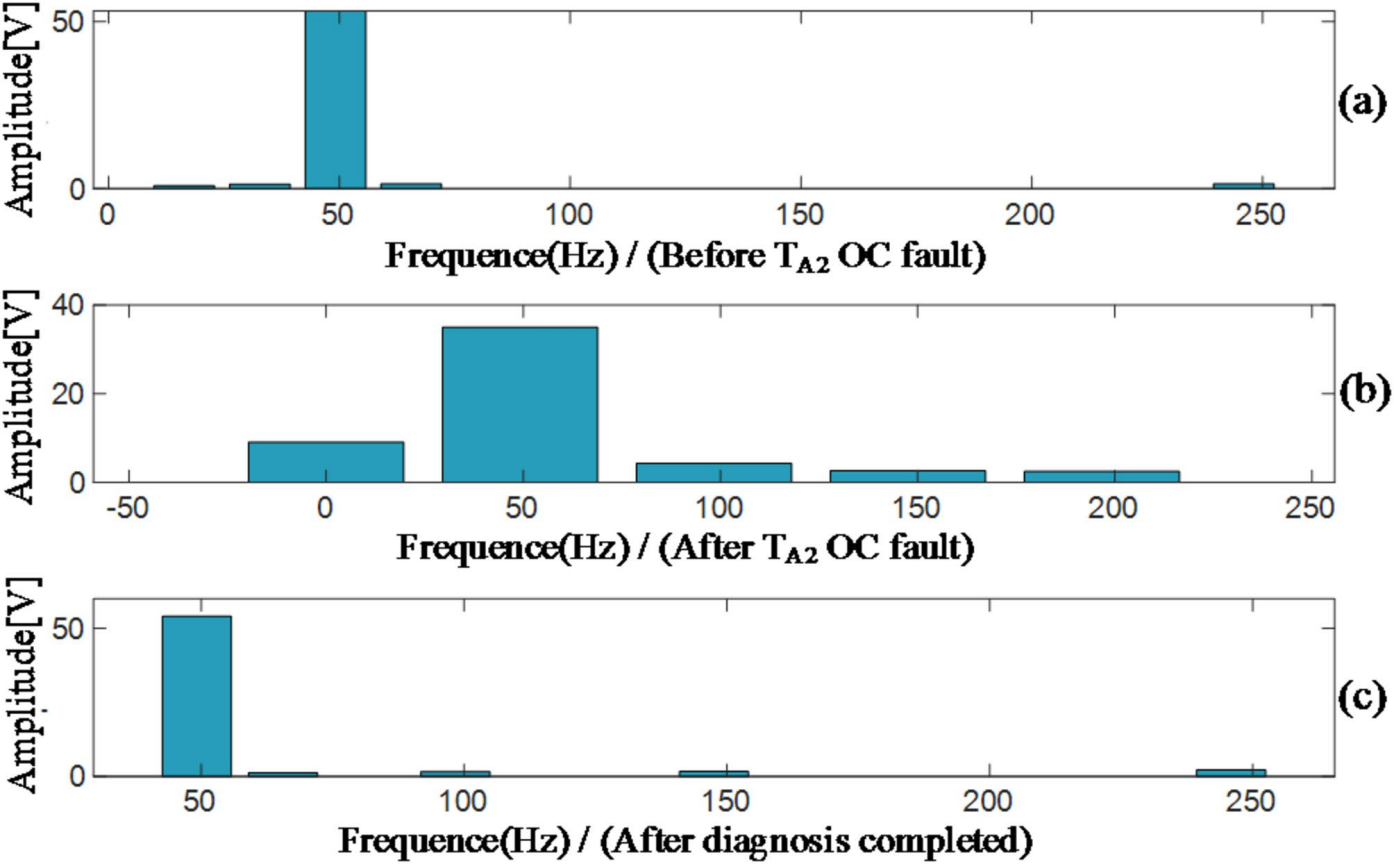
Fast Fourier decomposition results of voltage.

In Fig. 16, the three sub-figures a, b, and c represent the spectral diagrams of the line voltage obtained through Fast Fourier Transform analysis under three conditions: before the open-circuit fault of T_A2_, after the open-circuit fault of T_A2_, and after the proposed fault diagnosis algorithm is completed. As can be seen from Fig. 16 (a), before an OC fault of T_A2_, which represents normal operation, the harmonic content is very low. From Fig. 16 (b), it can be observed that after the open-circuit of T_A2_, the DC component, second-order harmonics, and third-order harmonics have increased significantly. The increase in the DC component can easily cause magnetic saturation in rotating electrical machines, leading to overheating and other phenomena, which severely threatens the service life of the motor. As shown in Fig. 16 (c), after fault diagnosis, the DC component and the second- and third-order harmonics have been significantly reduced.

Table 6 offers a comparative analysis of the diagnostic method presented in this study with other diagnostic methods mentioned in the introduction. The Tac in diagnostic time column specifies the fundamental wave period of the inverter’s output signal. Robustness indicates the strategy’s resilience against load disturbances, while applicable targets indicate whether the diagnostic strategy is tailored for two-level or three-level inverters. Upon comparison, it becomes evident that the proposed fault diagnostic method exhibits superior performance in terms of diagnosis speed, accuracy, robustness, and several other facets.

**Table 6:** Comparison with other diagnosis methods.

Methods	Diagnosis time	Accuracy	Robustness	Applicable objectives	Additional hardware
Ref 11	0.1Tac-1Tac	Medium	High	Three-level inverter	No
Ref 13	0.2Tac-0.7 Tac	High	High	Two-level inverter	No
Ref 18	0.5Tac-0.7Tac	High	High	Three-level inverter	No
Ref 19	0.5Tac-1Tac	High	Low	Three-level inverter	No
Ref 20	0.8Tac-1.5Tac	High	High	Three-level inverter	No
Ref 21	0.08Tac-1.58Tac	High	High	Three-level inverter	No
The proposed method	0.05Tac-0.5Tac	High	High	Three-level inverter	No

## Conclusion

A novel dual-mode voltage residual model-based diagnostic strategy has been developed for OC fault detection in T-type three-level inverters, enabling rapid fault localization and enhanced system reliability. The strategy accurately identifies faults by analyzing the residual values of line voltages in both normal mode and FTC mode. Experimental results have shown that the strategy offers benefits in terms of diagnostic speed, robustness, and harmonic suppression. By leveraging the three-level FTC algorithm, the harmonics generated during the diagnostic process is minimized, thereby facilitating the continuous and dependable operation of the inverter. While the proposed diagnostic strategy is applicable across all modulation modes of T-type three-level inverters, its diagnostic threshold remains fixed. We have set a high threshold to avoid misdiagnosis, which may result in longer fault detection times.

In future work, sampling error, dead time, and real-time current magnitude could be integrated into the threshold design to allow for real-time adjustments based on operational conditions, thus further improving reliability and diagnostic speed. It’s notable that the proposed fault diagnosis algorithm integrates a three-level FTC algorithm. The FTC algorithm is established on the foundation of SVPWM control. So, the current fault diagnosis algorithm is specifically tailored for multi-level inverters employing SVPWM control. It is effective for various multi-level inverters, including midpoint-clamped and flying capacitor types, which utilize SVPWM control. However, the diagnostic algorithm is not applicable to two-level inverters due to their lack of redundant switches.

## Data Availability

All data generated and analyzed during the current study are available from the corresponding author on reasonable request.

## References

[1] Rodriguez, J., Bernet, S., Wu, B., Pontt, J. O. & Kouro, S. Multilevel voltage-source-converter topologies for industrial medium-voltage drives.* IEEE Trans. Ind. Electron.*** 54**(6), 2930–2945. 10.1109/TIE.2007.907044 (2007).

[2] Schweizer, M. & Kolar, J. W. Design and implementation of a highly efficient three-level T-type converter for low-voltage applications. *IEEE Trans. Power Electron.* **28** (2), 899–907. 10.1109/TPEL.2012.2203151 (2013).

[3] Poorfakhraei, M., Narimani, A. & Emadi A review of multilevel inverter topologies in electric vehicles: current status and future trends. *IEEE Open. J. Power Electron.* **2**, 155–170 (2021).

[4] Huang, Z., Zhou, D., Wang, L., Shen, Z. & Li, Y. A review of singlestage multiport inverters for multisource applications. *IEEE Trans. Power Electron.* **38** (5), 6566–6584 (2023).

[5] Xu, S., Zhang, J. & Hang, J. Investigation of a fault-tolerant threelevel T-type inverter system.* IEEE Trans. Ind. Appl.*** 53**(5), 4613–4623 (2017).

[6] Wang, Z., Li, Z., Bai, P. T., Krein & Ma, H. A voltage vector residual Estimation method based on current path tracking for T-type inverter open-circuit fault diagnosis. *IEEE Trans. Power Electron.* **36** (12), 13460–13477. 10.1109/TPEL.2021.3087488 (2021).

[7] Zhou, S., Zhou, L. & Sun, P. Monitoring potential defects in an IGBT module based on dynamic changes of the gate current. *IEEE Trans. Power Electron.* **28** (3), 1479–1487. 10.1109/TPEL.2012.2210249 (2013).

[8] Liu, X. et al. Optimal current ripple PWM for three-level inverter with common mode voltage reduction. *IEEE Trans. Industr. Electron.* **69** (5), 4890–4900. 10.1109/TIE.2021.3080199 (2022).

[9] Zhang, W. & He, Y. A hypothesis method for T-type three-level inverters open-circuit fault diagnosis based on output phase voltage model. *IEEE Trans. Power Electron.* **37** (8), 9718–9732. 10.1109/TPEL.2022.3151731 (2022).

[10] Liang, Y., Wang, R. & Hu, B. Single-switch open-circuit diagnosis method based on average voltage vector for three-level T-type inverter. *IEEE Trans. Power Electron.* **36** (1), 911–921. 10.1109/TPEL.2020.3003058 (2021).

[11] Song, L. et al. Characteristics of common-mode voltage offset in small sectors and Oc fault diagnosis method for three-level inverter. 10.1007/s11668-023-01826-1 (2023).

[12] Chen, T. & Pan, Y. A novel diagnostic method for multiple open-circuit faults of voltage-source inverters based on output line voltage residuals analysis. *IEEE Trans. Circuits Syst. II Express Briefs*. **68** (4), 1343–1347. 10.1109/TCSII.2020.3034492 (2021).

[13] Cai, R. et al. Single-switch open-circuit diagnosis method based on 3D vector shift for T-type three-level inverters. *J. Power Electron.* **25**, 181–192. 10.1007/s43236-024-00879-1 (2025).

[14] Gong, Z., He, X. & Han, P. Diagnosis of open circuit faults for three-phase three-level converters based on the change rate of current residual. In *IEEE 16th Conference on Industrial Electronics and Applications (ICIEA), Chengdu, China, 2021*, 861–866. 10.1109/ICIEA51954.2021.9516070 (2021).

[15] Huang, W. et al. Sept., Current-based open-circuit fault diagnosis for PMSM drives with model predictive control.* IEEE Trans. Power Electron.* **36**(9), 10695–10704. 10.1109/TPEL.2021.3061448 (2021).

[16] Wang, X., Feng, T., Sun, Z., Wang & Cheng, M. Relative β-axis residual voltage signal based fault detection for inverter switch open-circuit failure. * IEEE Trans. Power Electron.* **38**(9), 11315–11326. 10.1109/TPEL.2023.3283580 (2023).

[17] An, Q. T., Sun, L. & Sun, L. Z. Current residual vector-based open-switch fault diagnosis of inverters in PMSM drive systems. *IEEE Trans. Power Electron.* **30** (5), 2814–2827. 10.1109/TPEL.2014.2360834 (2015).

[18] Shi, L. et al. A fault diagnosis method for T-type three-level topology APF. *Electr. Machines Control* **26**(02):94101. 10.15938/j.emc.2022.02.010 (2022).

[19] Yuan, Z., Shi, L., Wen, C. & Han, L. A fault detection method for T-type inverters based on voltage residual. *Power Electron. Technol.* **56** (11), 4–7 (2022).

[20] Ma, X., Li, M., Wei, S. & Shi, H. Diagnosis of open -circuit fault in T-type inverter. *Trans. China Electrotech. Soc.* **33** (10), 2324–2333. 10.19595/j.cnki.1000-6753.tces.170137 (2018).

[21] Zhang, W., He, Y. & Chen, J. A robust open-circuit fault diagnosis method for three-level T-type inverters based on phase voltage vector residual under modulation mode switching. *IEEE Trans. Power Electron.* **38** (4), 5309–5322. 10.1109/TPEL.2022.3230091 (2023).

